# Measuring sensitivity to social distancing behavior during the COVID-19 pandemic

**DOI:** 10.1038/s41598-022-20198-4

**Published:** 2022-09-29

**Authors:** Constantine E. Kontokosta, Boyeong Hong, Bartosz J. Bonczak

**Affiliations:** 1 Marron Institute of Urban Management, New York University, 370 Jay Street, Brooklyn, 11201 NY USA; 2 Center for Urban Science and Progress, New York University, 370 Jay Street, Brooklyn, 11201 NY USA

**Keywords:** Epidemiology, Computer science, Statistics, Human behaviour

## Abstract

Social distancing remains an effective nonpharmaceutical behavioral interventions to limit the spread of COVID-19 and other airborne diseases, but monitoring and enforcement create nontrivial challenges. Several jurisdictions have turned to “311” resident complaint platforms to engage the public in reporting social distancing non-compliance, but differences in sensitivity to social distancing behaviors can lead to a mis-allocation of resources and increased health risks for vulnerable communities. Using hourly visit data to designated establishments and more than 71,000 social distancing complaints in New York City during the first wave of the pandemic, we develop a method, derived from the Weber-Fechner law, to quantify neighborhood sensitivity and assess how tolerance to social distancing infractions and complaint reporting behaviors vary with neighborhood characteristics. We find that sensitivity to non-compliance is lower in minority and low-income neighborhoods, as well as in lower density areas, resulting in fewer reported complaints than expected given measured levels of overcrowding.

## Introduction

Although more than 8.3 billion vaccine doses have been administered globally (as of December 2021)^1^, the COVID-19 pandemic continues due to the transmission characteristics of the SARS-CoV-2 virus^2–6^, the absence of definitive treatment^7^, and the emergence of more contagious variants^8^. Based on prior pandemic experience, public health experts have turned to well-established nonpharmaceutical interventions (NPI), such as social distancing and the use of face coverings, to limit exposure risk from close contacts with infected individuals^9–13^. Studies have shown that these behavioral changes are effective in reducing the reproduction number ($R_0$) and slowing the dispersion of COVID-19 infections^5,6,12,14,15^.

Despite government-imposed public health restrictions and recommendations, cognitive and behavioral responses to social distancing guidelines—both mandatory and voluntary - are inconsistent across different sociocultural and political communities^6,16–18^. Even in areas with similar policy frameworks, studies have found that disparities in social distancing compliance and behavior change can result from varying belief systems, social capital and cohesion, and social norms, associated with neighborhood demographic, socioeconomic, and political attributes^13,19–34^. For instance, communities where a majority of residents identify their political affiliation as Republican have been found to be less concerned about the virus and its potential health effects, and therefore tend to be less likely to engage in self-protective behaviors, including mask wearing, compared to Democrat-leaning neighborhoods^26,29,32^. As individual behavioral responses are influenced by peers (e.g. family, friends, and neighbors), neighborhood social and cultural norms can reinforce cognitive biases leading to similar community outcomes^20,30,34^. For example, communities or countries with a stronger sense of collective identity are associated with greater support for social distancing behaviors^33,34^. Additionally, characteristics of neighborhoods and their residents impact the ability of households to change behaviors given specific circumstances. Higher-income households, for example, are more likely to be able to work from home, thus making it easier for residents in more wealthy neighborhoods to adhere to stay-at-home orders or other social distancing guidance^19,24,25^. Similarly, gender has been associated with the willingness to wear face coverings^34^. Consequently, individuals or households may have different comfort levels with social distancing compliance and mask wearing behaviors depending on what they see, what they believe, and what they experience in their communities. Empirically measuring these emotions and feelings, particularly at the neighborhood or community scale, creates nontrivial challenges for the design and enforcement of local health policy.

Resident-initiated reporting plays an important role in identifying and responding to neighborhood conditions and residents’ need for more effective and equitable city service delivery^35^. In New York City (NYC), new complaint types related to social distancing and face covering compliance were introduced to the NYC311 system—one of the largest e-government systems and a key co-production channel—on March 28, 2020, a week after the introduction of a statewide stay-at-home order. Residents have the ability to report - via phone, web, or text - observed or perceived violations to social distancing and mask wearing rules, thus providing public health and law enforcement agencies with a human surveillance network to identify real-time infractions. NYC311 collects these complaints along with detailed information on the report, including timestamp and geo-location, for further investigation and potential response. From March 28, 2020 through July 4, 2020, there were more than 71,000 social distancing complaints (both for social distancing and face covering non-compliance) reported to NYC311, equivalent to an average of approximately 480 per day (Fig. S1). During this period, social distancing complaints were one of the top three complaint types, preceded by noise and building issues (Fig. S2), suggesting a significant level of concern among NYC residents.

However, relying on 311 complaints to enforce social distancing rules introduces potential bias into observed compliance rates and enforcement actions. It is expected that individuals will not report social distancing concerns at the same rate, as residents’ socio-behavioral responses to *other’s* social distancing practices are sensitive to their own circumstances and neighborhood conditions. Therefore, when facing similar social distancing violations, 311 complaints may not present a complete and objective picture of social distancing compliance across a city. A similar concern has been found with other types of complaints, including a negative correlation between neighborhood conditions and complaint reporting^36^ and positive associations between a community’s level of trust in government and the propensity to report a problem^35,37–41^. Varying levels of sensitivity to social distancing violations result in inconsistent patterns of reporting and, potentially, an under- or over-representation of local problems and potential health risks. Therefore, fair and effective local public health policy necessitates an understanding of variations in perception and sensitivity to social distancing behaviors.

In this paper, we develop a method to quantify neighborhood social distancing sensitivity (*SDS*) to assess how tolerance of social distancing infractions and complaint reporting behaviors vary with neighborhood demographic, socioeconomic, and political characteristics and local health risk profiles. We focus on New York City (NYC) during the first wave of the pandemic, from March 28, 2020 through July 4, 2020. New York City provides a unique experimental environment to study social distancing sensitivity given its diverse neighborhoods, the magnitude of the first wave of COVID-19 disease spread, and its robust 311 service request system. We integrate and analyze NYC311 social distancing complaints and mobility data provided by SafeGraph, Inc., which include device visit counts for more than 87,000 Point of Interest (POI) locations. We geocode all social distancing complaints and POI data using a spatial database of NYC buildings mapped using a unique building identifier. Our methodology consists of three steps: First, we introduce a generalizable method for quantifying social distancing sensitivity at the neighborhood scale to absolute and relative changes in neighborhood activity levels using a psychophysical conceptual framework and the Weber-Fechner law to measure human responses to external stimuli. Second, we identify and analyze disparities in neighborhood sensitivity to social distancing and associated 311 reporting behavior by estimating local variations in the relative magnitude of crowding – above typical levels – leading to complaint reports. Lastly, we identify demographic, socioeconomic, political, and public health factors correlated with disparities in social distancing sensitivity. We also examine the relationship between the sensitivity metric and other health behaviors and outcomes, including local vaccination rates, COVID-19 case rates, and police response to complaints. Our findings provide insight into systemic bias in resident reporting and the community characteristics impacting socio-behavioral responses to public health interventions. As such, our work is intended to support equitable and targeted public health policies that overcome current constraints to the objective and timely evaluation of local decision-making processes.

## Results

### Measuring sensitivity to changes in POI activity intensity as a proxy for social distancing

We assume that the number of visits to a POI during a given time period is a proxy for the intensity of social activity at that location. We also assume that a 311 social distancing complaint represents the behavioral response to the external stimuli of observed overcrowding, relative to reference activity levels (when a complaint is not reported). A complaint is reported when a threshold level of human density, as determined by the resident reporting the complaint, violates acceptable social distancing behavior for a particular community. We consider the hypothesis that higher POI visit density (visits per square foot of POI space), compared to "normal" levels (and holding POI floor area constant), is more likely to result in a social distancing complaint. In the absence of socio-spatial disparities in the behavioral response to perceived social distancing non-compliance, the rate of complaints should be stable across neighborhoods given similar relative activity levels.

**Figure 1:**
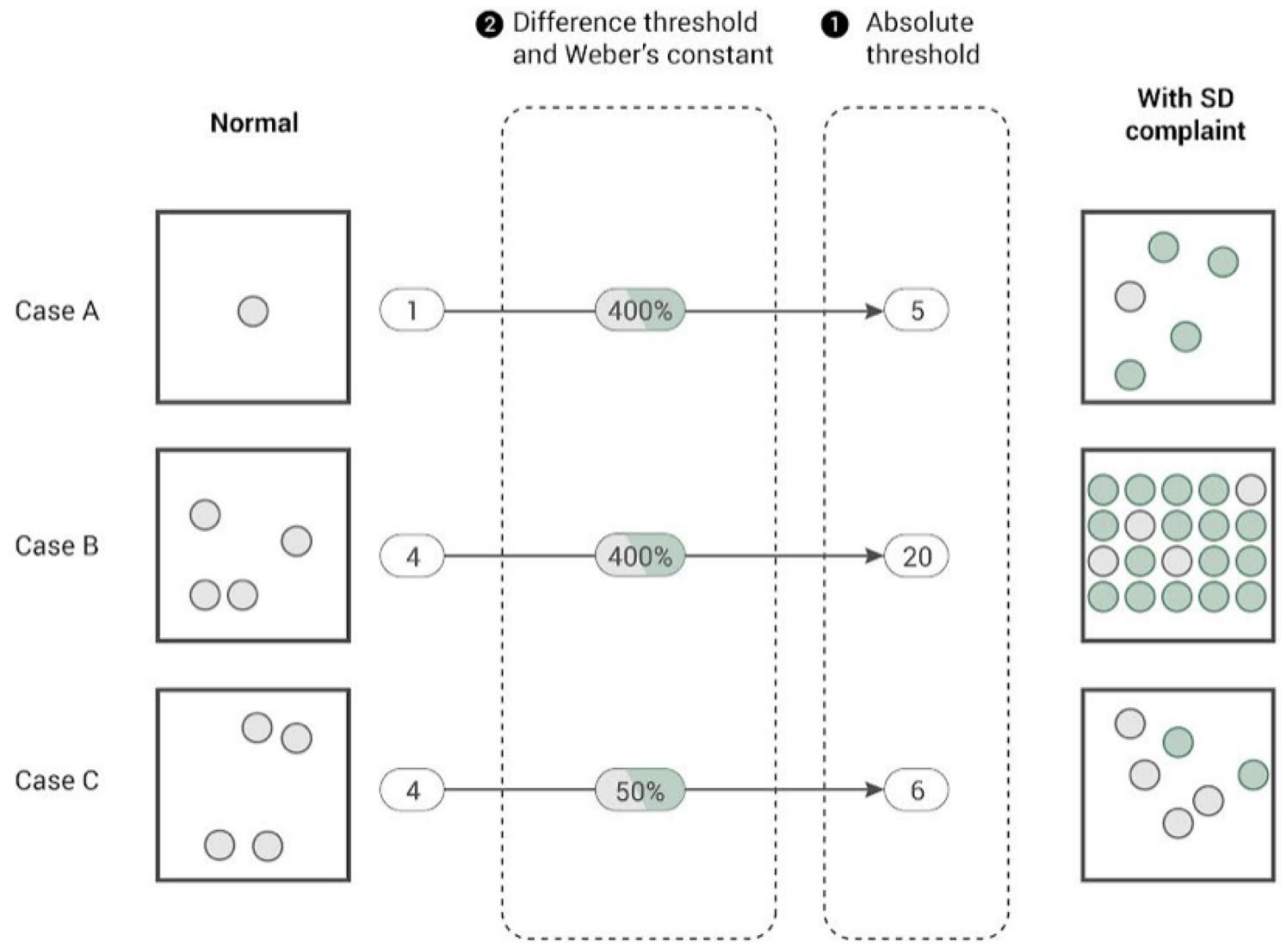
The conceptual framework for absolute and difference thresholds for social distancing violation reporting. We compare normal levels of social activities (when a complaint is not reported) to the external stimuli of observed overcrowding (when a complaint is reported).

To test this hypothesis, we introduce social distancing sensitivity (*SDS*) as a location-specific measure of the response to an external stimuli, in this case perceived changes in crowding above the complainant’s expectation for appropriate and safe social distancing behavior. The sensitivity measure is derived from the psychophysical concepts of absolute and relative sensory thresholds stemming from *Weber’s Law*^42,43^, which has been used to evaluate perceptions of the severity of the COVID-19 pandemic^44,45^. The absolute threshold (*A*) is defined as the minimum stimulus required to result in a reaction, while a relative or difference threshold (*D*, also referred to as a *Just Noticeable Difference threshold*) is the minimum increase of a stimulus needed to produce a perceptible change in sensation relative to the existing level of stimulus (see Methods for details)^45,46^. In this study, we quantify the absolute activity intensity for each POI by calculating the number of POI visits per square foot (POI visit density) for six-hour time windows over the study period. The difference threshold for each POI is quantified by estimating the percent difference between normal POI visit density (when no complaints are reported) and POI visit density when a complaint is reported.

Figure 1 represents the absolute and difference thresholds in the social distancing context. For example, if two POIs (Case B and C) have 20 and 6 visits, respectively, when a social distancing complaint is reported, the response to the latter POI is considered more sensitive than the former with respect to the absolute threshold of crowding, controlling for other factors (e.g. POI area and time period of the observation). Furthermore, if both POIs have four baseline visits (when no complaint is reported) for that time period, the difference threshold constants are 400% ($=(20-4)/4$) and 50% ($=(6-4)/4$), again reflecting the higher sensitivity in response to conditions in Case C (Fig. 1). Social distancing sensitivity is expressed as a 0 to 100 score and can be defined mathematically as the inverse normalization of the *MinMax* re-scaled subjective sensation (*SS*) of POI visit density when a social distancing complaint is reported, accounting for both the absolute threshold (*A*) and the difference threshold (*D*) based on *Fechner’s Law*^45,47^. Therefore, sensitivity is specified as:

1 $$\begin{aligned}&\text {Social Distancing Sensitivity }(SDS) = \left| 1 - \frac{SS-min(SS)}{max(SS)-min(SS)} \right| \times 100 \end{aligned}$$

2 $$\begin{aligned}&\text {Subjective Sensation }(SS) = D \times log A \end{aligned}$$

where *SDS* is the sensitivity measure for social distancing non-compliance (0: least sensitive, 100: most sensitive), *SS* is the subjective sensation, *D* is the difference threshold constant, and *A* is the absolute threshold (see Methods for detailed descriptions of the data and methodology). As a result, a POI with a high *SS* is considered as a less sensitive case, while a POI with a low *SS* is identified as a more sensitive case.

**Figure 2:**
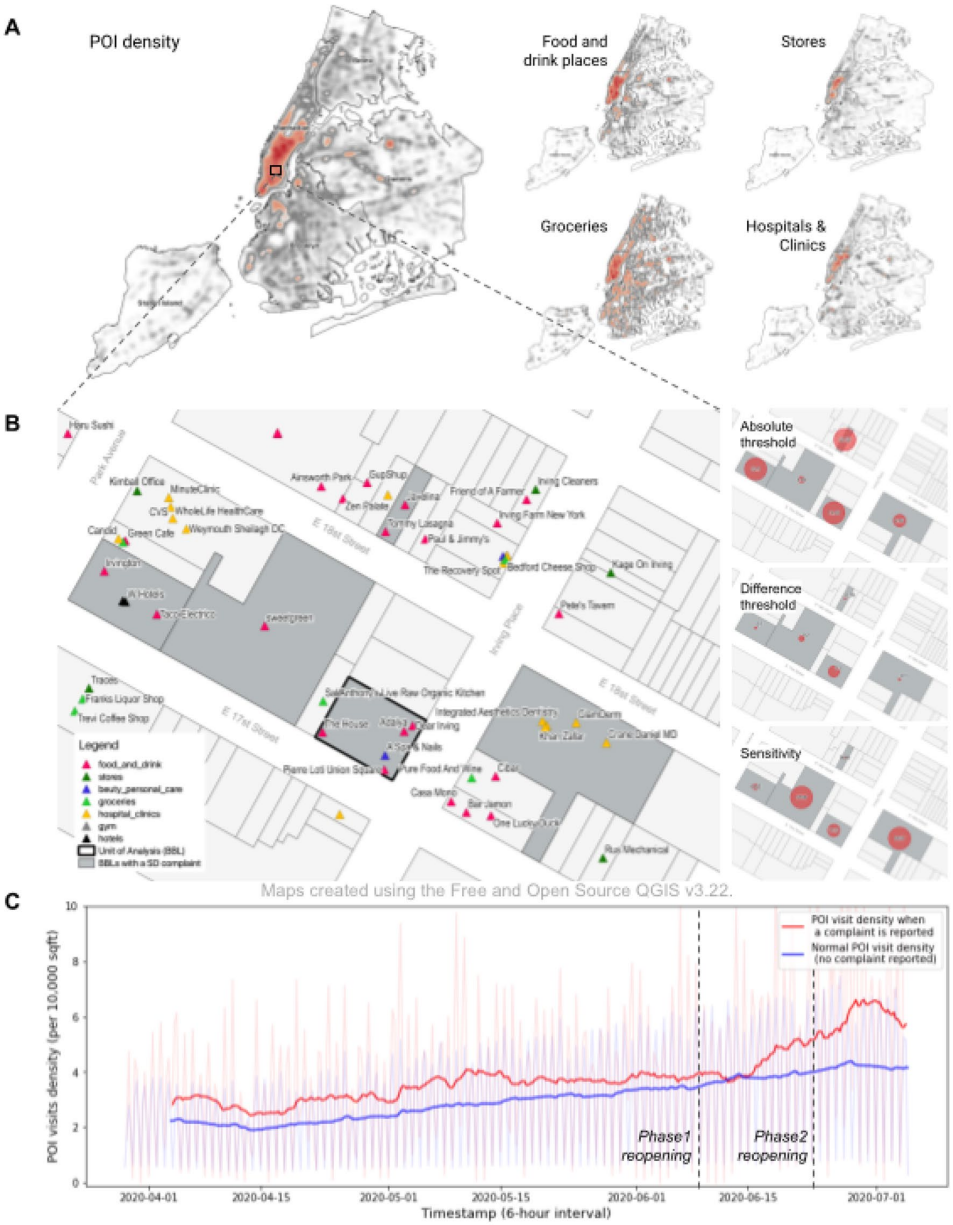
(**A**) POI density in New York City. Maps show the top four most common POI types (food and drink places, stores, grocery stores, and hospital & clinics). (**B**) POI level social distancing sensitivity (*SDS*) measurement by building tax lot (BBL). We measure 7670 BBLs in NYC with one or more POIs where at least one social distancing complaint was reported (represented in grey). (**C**) Citywide 6-hour average POI visit density over the time period when a complaint was reported compared to a baseline.

**Table 1:** Descriptive statistics of POI-level sensitivity measurements by establishment type. This table is sorted by sensitivity (descending) and the absolute threshold unit is the number of visits per 10,000 sq.ft. of POI floor area for each six-hour time interval. Mean values are shown with standard deviations in parentheses.

POI type	Complaint volume	Mode hour of complaints	Absolute threshold (*A*)	Difference threshold *(D)*	Social Distancing Sensitivity score *(SDS)*
Hospitals / clinics	7,570 (22.1%)	14	4.32 (6.12)	0.16 (0.61)	69.06 (17.12)
Day care / schools	4343 (12.7%)	17	2.96 (4.16)	0.19 (0.76)	69.04 (16.26)
Stores	9606 (28.1%)	16	5.06 (8.49)	0.16 (0.63)	68.99 (17.56)
Beauty or personal care	5660 (16.6%)	16	5.06 (6.89)	0.17 (0.61)	68.54 (17.45)
Gym	3634 (10.6%)	16	5.46 (13.93)	0.20 (0.69)	68.36 (17.19)
Food and drink places	17,028 (49.8%)	18	9.19 (14.49)	0.19 (0.69)	67.95 (19.39)
Religious facilities	1074 (3.1%)	11	4.64 (9.07)	0.20 (0.68)	67.89 (18.24)
Banks / finance	5320 (15.6%)	14	4.62 (6.81)	0.22 (0.61)	67.58 (17.00)
Grocery stores	8676 (25.4%)	16	6.22 (9.31)	0.23 (0.62)	67.00 (17.51)
Parks / cultural facilities	3035 (8.9%)	17	7.47 (20.59)	0.29 (0.99)	66.63 (19.87)

After data processing and cleaning as described in the Materials and Methods section, we analyzed 7,670 buildings in NYC (mapped as tax lots based on the borough-block-lot or BBL identifier) with one or more POIs where at least one social distancing complaint was reported during the study period (hereafter labeled as “POI”). Citywide POI density and spatial distribution by BBL are presented in Fig. 2. POIs are spatially concentrated in higher-density areas of NYC, including Manhattan, the Bronx, downtown Brooklyn, and areas along major roadways in Queens and Staten Island, where commercial and mixed-use zoning is common. Grocery stores are relatively evenly distributed across the city, compared to other types of establishments. The largest group of 311 social distancing complaints (17,028, 49.8%) are associated with food and drink places, such as restaurants, cafes, or bars, followed by retail stores (9606, 28.1%), grocery stores (8,676, 25.4%), and hospitals and clinics (7570, 22.1%), as shown in Table 1. We also observe differences in the mode hour of complaints. For instance, complaints from food and drink places tend to be reported around 6pm in the evening, while the mode hour of complaints for grocery stores is 4 pm. On the other hand, religious facilities and hospitals/clinics have more complaints at earlier hours (11 am and 2 pm, respectively). This reflects varying visitation times by establishment type.

Figure 2C illustrates the citywide average number of POI visits per 10,000 square feet of space and six-hour time interval, for both when a social distancing complaint was reported and a baseline level when no complaint was reported over the study period. The trend line for when a complaint was reported (red) clearly shows higher POI visit density compared to "normal" POI visit density (blue). The time-series of POI visit density when a complaint was reported represents the absolute threshold (*A*), the minimum stimulus resulting in perception of overcrowding and the associated response of complaint reporting. The distance between the two lines illustrates the difference threshold (*D*), the minimum increase of POI visit density leading to a perceptible increase of sensation relative to normal POI activity. Citywide, the POI-level mean absolute threshold was 7.58 [95% CI of 7.29–7.87] (visits per 10,000 square feet) and the mean difference threshold was 0.18 [95% CI of 0.16–0.20], indicating that an increase in visit density of 18% was sufficient to perceive a violation of social distancing norms. We observed that POIs located in communities in Manhattan (below 125th street), downtown Brooklyn, and Williamsburg had lower absolute and difference thresholds, reflected in higher *SDS* values for these neighborhoods when compared to lower-density communities. POI-level sensitivity measurements provide the most granular estimates of disparities in *SDS*. For example, POIs on the Upper West Side of Manhattan (UWS) had lower absolute and difference thresholds associated with higher *SDS* compared to POIs in the Borough Park neighborhood of Brooklyn. This suggests that residents of the UWS were more likely to report a social distancing complaint holding changes in visit density constant.

Table 1 presents descriptive statistics for POI-level *SDS* score estimates for the most common POI types. While difference thresholds and sensitivity are relatively consistent across the different establishment categories, we note that daycare/schools and hospitals/clinics had the highest *SDS* score values (69.06 and 69.04, respectively). Given the nature of these facilities and their visitors, the higher sensitivity is likely a result of greater caution about social distancing in the presence of vulnerable populations. Conversely, food and drink establishments and grocery stores showed relatively lower *SDS* scores (67.95 and 67.00, respectively) among the POI categories. These two POI types are often considered essential businesses that remained open during the outbreak and retained more regular, pre-COVID visit patterns than other POIs. We presume that people may have become accustomed to overcrowding at restaurants and while at the grocery store, but the necessity of visiting such places resulted in less sensitivity to social distancing infractions. Additionally, parks and cultural facilities with relatively large open spaces showed the lowest *SDS* score (66.63). There were fewer restrictions and capacity limits on parks and open spaces due to the lower risk of infection in outdoor settings, resulting in lower sensitivity to social distancing.

**Figure 3:**
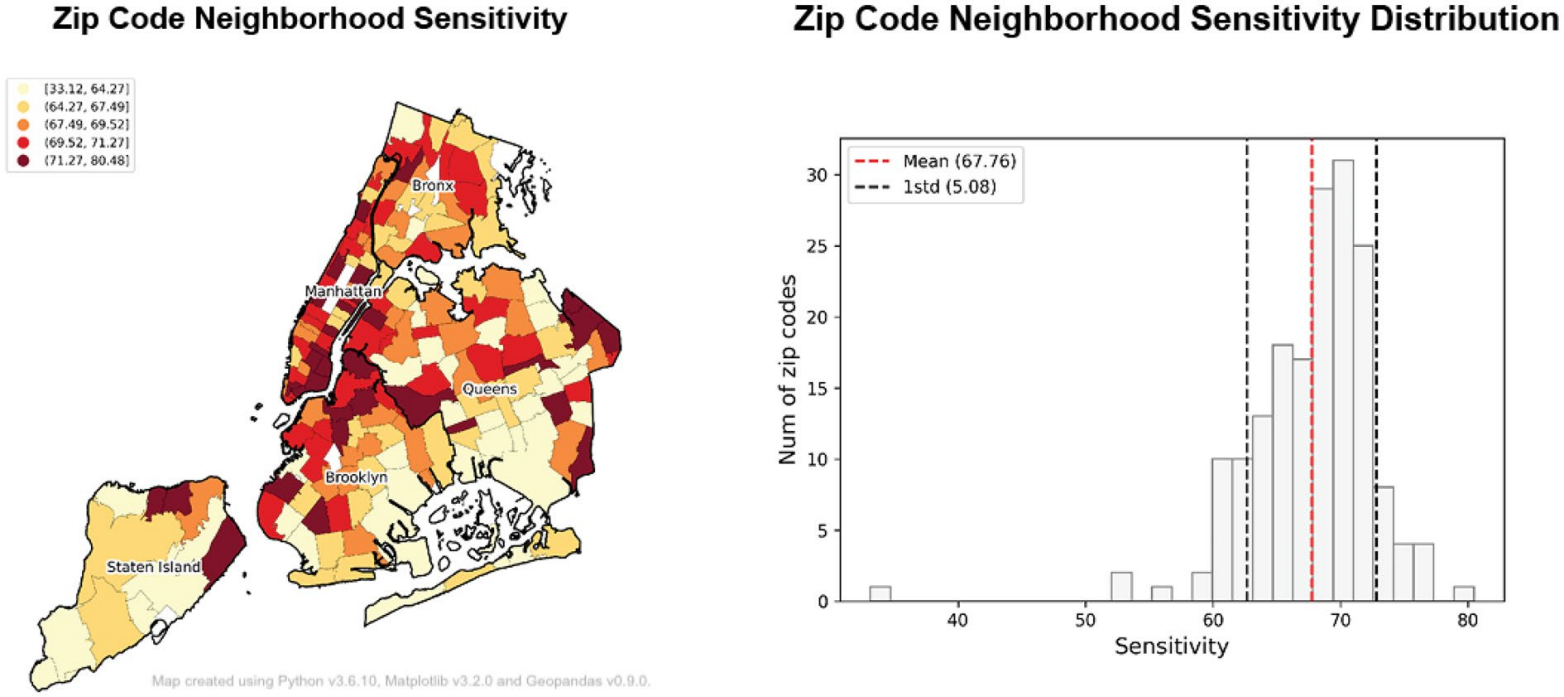
Zip code level sensitivity measurements.

### Neighborhood disparities in behavioral responses to social distancing

To examine neighborhood disparities in social distancing perception and reporting behavior, POI-level sensitivity measurements were aggregated to zip codes and joined with ancillary data. Figure 3 visualizes the spatial patterns of sensitivity across zip code neighborhoods. There are significant disparities in neighborhood social distancing sensitivity across the City, with a majority of neighborhoods in Manhattan, downtown Brooklyn, Williamsburg, and several in Queens presenting significantly higher sensitivity values. On the other hand, neighborhoods in South and Central Brooklyn, Queens, and Staten Island showed higher activity thresholds resulting in relatively low responsiveness to crowding. Therefore, fewer social distancing complaints were reported in less sensitive neighborhoods despite similar relative changes in visit density. This suggests that more sensitive neighborhoods may over-report social distancing violations even when neighborhood activity levels and social distancing behaviors are comparable.

**Figure 4:**
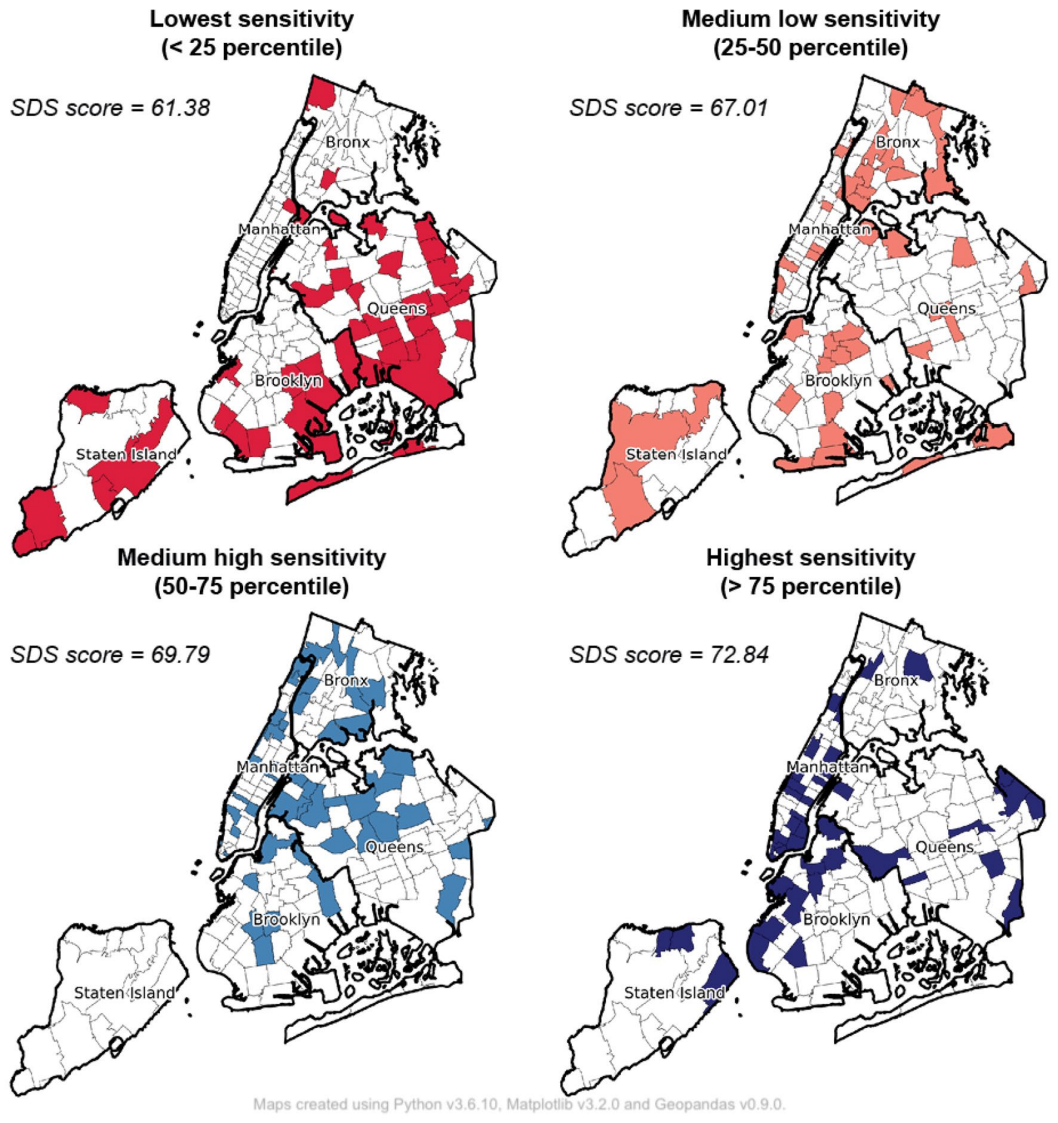
Neighborhood clusters by social distancing sensitivity (*SDS*) score.

Next, we explored associations between disparities in observed behavioral responses and neighborhood demographic, socioeconomic, and political characteristics. In order to contextualize neighborhood social distancing sensitivity and reporting behaviors, additional data resources were collected and integrated to incorporate a range of explanatory variables, including age, race, education, occupation, household type, and political affiliation (as described in the Materials and Methods section). Zip code neighborhoods were classified into quartiles based on the distribution of *SDS* values. Figure 4 presents spatial patterns and descriptive statistics for each of the four neighborhood groups. The least sensitive group, with scores below the 25th percentile, consists of 44 neighborhoods with a neighborhood mean *SDS* score of 61.38. These neighborhoods are spatially concentrated in central and south Brooklyn, Queens, and Staten Island. The most sensitive group (sensitivity scores above the 75th percentile) includes 44 neighborhoods with a mean *SDS* score of 72.84, most of which are located in Manhattan, Downtown Brooklyn, and Williamsburg. The remaining 88 neighborhoods are classified as either medium-low or medium-high sensitivity with mean *SDS* scores of 67.01 and 69.79 respectively.

We present neighborhood cluster characteristics in Table 2. Neighborhoods in the most sensitive group (*SDS* $$score \ge 75th \space percentile$$) had the highest proportion of non-Hispanic White population (54.87%), the smallest average household size (2.43), the highest median incomes ($89K, which is $21K higher than the least sensitive group) and educational attainment (28.34% with Bachelor’s degree), and a significantly higher proportion of housing units valued over $750K (45.63%). Additionally, neighborhoods in this group had a greater percentage of employees working in professional, scientific, and management occupations (17.72%) and relatively more employees working from home (6.02%) compared to other groups. Residents who work from home have a statistically significant positive impact on social distancing sensitivity and reporting likelihoods. Since higher income employees in professional occupations are more likely to be able to work remotely and thus shift toward working from home^48^, these groups could significantly change mobility behaviors and thus reduce potential interactions with others, reinforcing social norms that emphasize social distancing compliance in the community. Also, another distinct feature of these neighborhoods is high population density associated with higher-density built environments. As higher densities correlate with greater infection risk resulting from increased probability of close contacts, residents who live in higher density communities may pay more attention to social distancing compliance, leading to higher sensitivity and increased reporting of non-compliance.

**Table 2:** Descriptive statistics of neighborhood groups by social distancing sensitivity (*SDS*) quartile.

Feature	*SDS* < 25th percentile	*SDS* 25th–50th percentile	*SDS* 50th–75th percentile	*SDS* > 75th percentile	ANOVA F-stats
Population density	25470.86 (20670.26)	43736.60 (27920.76)	53297.03 (32060.62)	49689.38 (38252.25)	7.27***
Non-Hispanic White, %	0.41 (0.28)	0.42 (0.26)	0.48 (0.24)	0.55 (0.22)	2.90**
Black, %	0.24 (0.28)	0.28 (0.24)	0.18 (0.21)	0.15 (0.21)	2.56*
Median income $,	68,000 (22,000)	63,000 (33,000)	75,000 (44,000)	89,000 (41,000)	4.42***
College degree, %	0.19 (0.06)	0.21 (0.10)	0.25 (0.11)	0.28 (0.10)	8.71***
High school degree, %	0.29 (0.07)	0.24 (0.09)	0.21 (0.09)	0.17 (0.10)	14.16***
Housing units over 750K, %	0.20 (0.17)	0.28 (0.24)	0.35 (0.26)	0.45 (0.27)	9.11***
Average household size	2.93 (0.42)	2.63 (0.45)	2.56 (0.46)	2.43 (0.56)	8.53***
Owner-occupied units, %	0.51 (0.21)	0.32 (0.21)	0.27 (0.19)	0.35 (0.22)	11.25***
Gini index	0.46 (0.05)	0.49 (0.05)	0.48 (0.05)	0.50 (0.07)	5.08***
Unemployment rate, %	0.07 (0.04)	0.08 (0.03)	0.07 (0.03)	0.06 (0.03)	2.34*
Professional and scientific workers, %	0.11 (0.03)	0.13 (0.06)	0.15 (0.07)	0.18 (0.07)	11.50***
Healthcare workers, %	0.06 (0.03)	0.06 (0.04)	0.04 (0.04)	0.03 (0.03)	6.99***
Work from home workers, %	0.03 (0.01)	0.04 (0.02)	0.04 (0.02)	0.06 (0.05)	10.52***
No health insurance, %	0.08 (0.04)	0.08 (0.03)	0.08 (0.06)	0.06 (0.03)	2.84**
Private health insurance, %	0.60 (0.15)	0.58 (0.17)	0.62 (0.18)	0.69 (0.17)	3.86**
One or two family housing, %	0.55 (0.29)	0.26 (0.27)	0.18 (0.24)	0.23 (0.31)	16.32***
Office area, %	0.03 (0.02)	0.08 (0.11)	0.09 (0.15)	0.14 (0.23)	4.36***
Republican voters, %	0.62 (0.40)	0.49 (0.36)	0.40 (0.25)	0.41 (0.26)	4.25***
Non-profit organizations, per 1K residents	0.15 (0.16)	0.32 (0.93)	0.65 (1.65)	1.39 (3.20)	3.82**
COVID-19 case rate, per 10K residents	2649.09 (680.94)	2527.86 (904.65)	2256.11 (833.83)	1833.22 (907.53)	8.22***
Vaccination rate, %	0.66 (0.14)	0.62 (0.12)	0.70 (0.16)	0.74 (0.16)	5.33***

22 features with statistically significant differences between groups based on one-way ANOVA and Tukey’s multicomparison method (Table S2). Mean values are shown with standard deviations in parentheses. COVID-19 and vaccination features are based on data provided by the New York City Department of Health through July 2, 2020 and July 30, 2021, respectively. ***$p-value<0.01$, **$p-value<0.05$, *$p-value<0.1$.

Neighborhoods with larger proportions of minority populations and lower median incomes were less likely to be sensitive to social distancing violations, resulting in potential under-reporting of non-compliance that would trigger a complaint elsewhere. More specifically, neighborhoods in the least sensitive group (*SDS* $score \le 25th \space percentile$) are located in lower population density communities in Staten Island, the Bronx, Queens, and South Brooklyn. These neighborhoods were found to have the largest share of one- or two-family housing (54.97%), approximately 2.4 times higher than neighborhoods in the most sensitive group. Also, these neighborhoods had relatively larger average household size (2.93 persons), and a higher percentage of housing units occupied by owners (51.16%). This suggests that residents in these neighborhoods may have had relatively low sensitivity due to both lower built environment density and fewer visitors from outside of the respective communities, resulting in more localized social activities during the pandemic. When compared to other groups, the least-sensitive communities had higher proportions of Black population (24.46%), lower median incomes (68K), lower housing prices (20.22% of housing units valued above 750K), lower educational attainment (18.72% with a Bachelor’s degree), and higher unemployment rates (6.97%). Compared to other communities, a greater share of employees in this neighborhood group worked in healthcare support occupations (6.08%). Employees who work in occupations deemed essential services were required, in most cases, to maintain typical mobility behaviors, particularly commuting patterns. Residents in neighborhoods where the proportion of essential workers is higher, therefore, may become de-sensitized to social distancing violations, resulting in lower sensitivity values and, by extension, under-reporting. Overall, we found that economically disadvantaged and vulnerable neighborhoods have lower sensitivity, and thus tend to under-report social distancing non-compliance, which aligns with social distancing behaviors found in previous studies^6,19,24,25^. Our results highlight the significant equity concerns in reporting-based enforcement programs. Vulnerable communities face greater health risks both in terms of potential exposures and severity of disease after infection, while observed under-reporting may lead to an under-allocation of public health resources to neighborhoods in need.

Next, we explore the relationship between political affiliation and sensitivity at the neighborhood scale, building on previous studies of the influence of political ideology on attitudes toward social distancing^26,29,32,33^. We find that communities with a higher percentage of residents who voted for Donald J. Trump in the 2020 Presidential election were less likely to report social distancing violations. There are many possible explanations for this relationship. One possibility is that Republican-leaning communities tend to downplay the significance of COVID-19 as a health risk and underestimate the severity of the pandemic compared to neighborhoods with more voters affiliated with the Democratic party, resulting in fewer changes in behavior, including adherence to social distancing guidelines. This corresponds with lower collective sensitivity to social distancing non-compliance and a lower likelihood of 311 reporting. Another explanation suggests there may be varying levels of trust in government and government agencies across the political spectrum. For Republican-leaning communities in NYC, where the governor of the State and the mayor of the City represented the Democratic party during the study period, their residents may be less willing to interact with government and report other residents’ behavior through the e-government system. We also found that there is a positive association between social distancing sensitivity and measures of social infrastructure. We used the number of non-profit community organizations per 1000 residents in each zip code neighborhood to evaluate the influence of social cohesion on social distancing compliance. There were, on average, 0.15 non-profit organizations per 1000 residents in the least sensitive neighborhoods, compared to 1.39 in the most sensitive neighborhoods. If we assume this social infrastructure measure can be used as a proxy for social cohesion within a community, this finding suggests communities with higher social cohesion may reinforce positive socio-cultural norms around compliance with COVID-19 restrictions, resulting in higher sensitivity to social distancing non-compliance and a higher likelihood of reporting.

**Table 3:** Neighborhood comparison: Descriptive statistics of POI-level sensitivity measurements for food and beverage places in a high sensitivity neighborhood (Park Slope) and a low sensitivity neighborhood (Bensonhurst).

Zip code	Neighborhood	Food and Drink Places		
		Absolute threshold (*A*)	Difference threshold *(D)*	Social distancing sensitivity (*SDS*)
11215	Park Slope	10.23	0.23	71.77
11214	Bensonhurst	15.93	0.39	64.89

**Figure 5:**
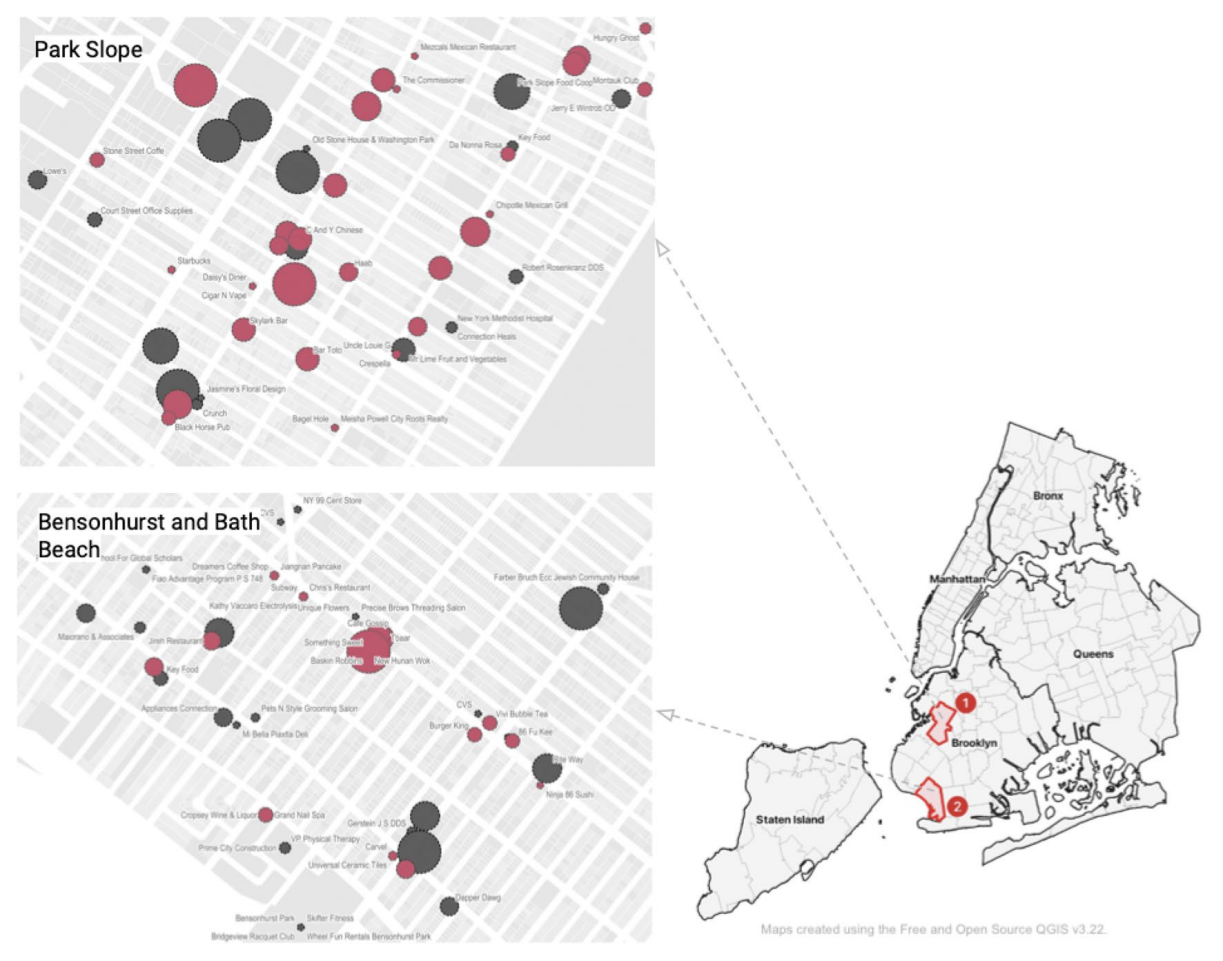
Case study: neighborhood comparison of sensitivity. Marker size represents relative sensitivity measurements. Left: Zip Code 11215, Park Slope area. Right: Zip Code 11214, Bensonhurst and Bath Beach area. Red markers represent food and drink places (e.g. restaurants, fast foods, cafes, or bars) and the grey markers represent other types of POIs.

To illustrate our findings, we select two predominantly residential neighborhoods representing the higher and lower sensitivity groups described in Table 3 and Fig. 5. The wealthy enclave of Park Slope in Brooklyn (zip code 11215) represents a higher sensitivity neighborhood, with approximately 80% of the population identifying as non-Hispanic White and a median household income of $124K. The lower sensitivity neighborhood, also in Brooklyn, is the Bensonhurst and Bath Beach area, a lower-income, working class community further from the Central Business Districts of Midtown and Lower Manhattan. In addition to socioeconomic differences, the percentage of voters who voted for Donald J. Trump in the 2020 Presidential election in Park Slope was just 19.20%, while more than 97% of Bensonhurst voters selected the Republican candidate. For comparison, in Park Slope, the average POI-level absolute threshold, difference threshold, and sensitivity for food and beverage places is 10.23, 0.23, and 15.38, respectively. Therefore, in general, a social distancing complaint was reported when observed overcrowding was 23% more than typical activity for that time period. On the other hand, the *SDS* score of 64.89 in Bensonhurst/Bath Beach indicates that a social distancing complaint was only reported if the increase in visitor density to food and beverage establishments exceeded 39%, on average.

### Linking social distancing sensitivity and public health outcomes

**Figure 6:**
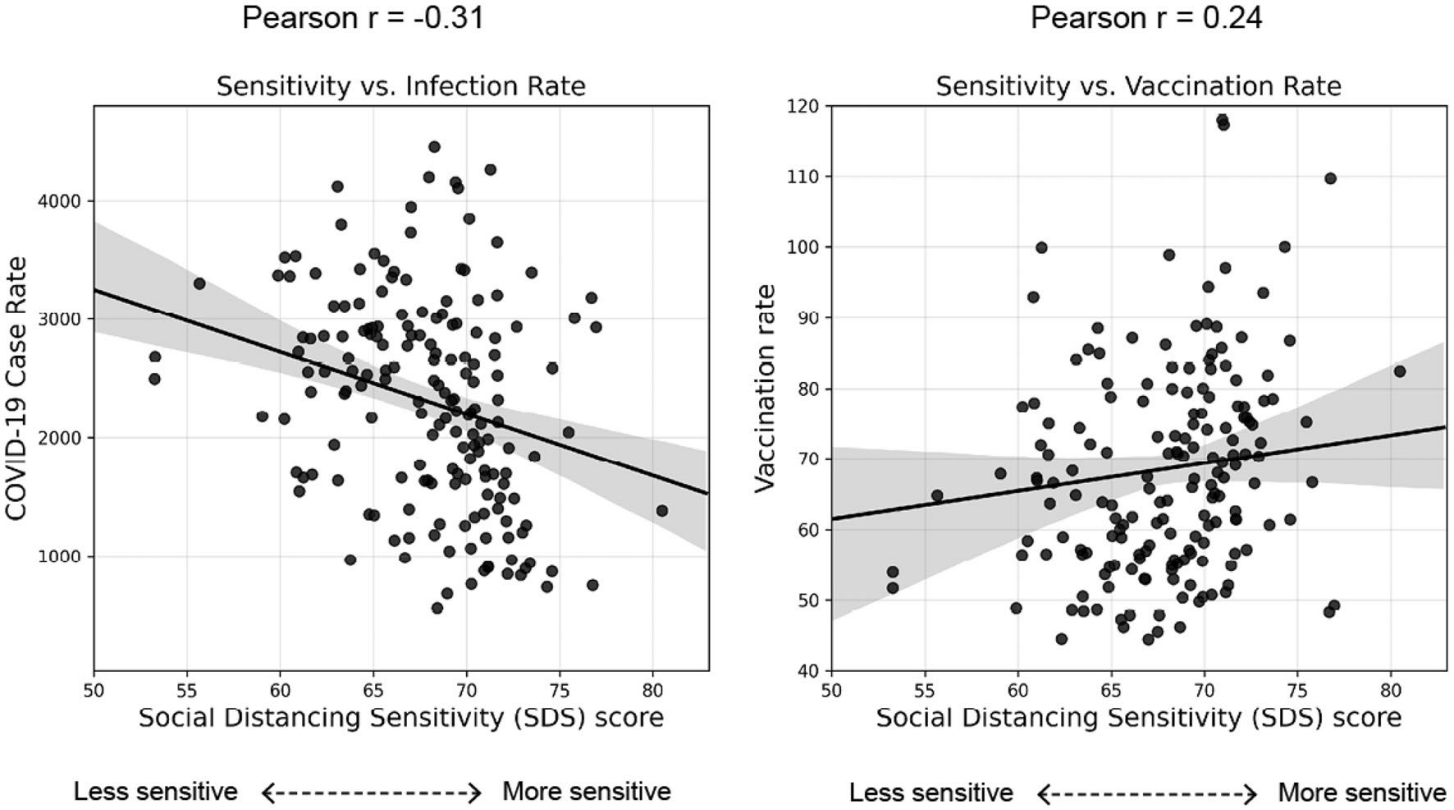
Relationship between neighborhood sensitivity and public health measures. Left: neighborhood sensitivity versus cumulative COVID-19 case rate as of July 2, 2020. Right: neighborhood sensitivity versus cumulative COVID-19 vaccination rate as of July 30, 2021.

We assume that our social distancing sensitivity metric represents the relative importance placed on social distancing behaviors in different communities. As such, it should follow that *SDS* is associated with other measures of public health outcomes and risk. To test this hypothesis, we examined neighborhood level cumulative COVID-19 infection rates as of July 2, 2020 and COVID-19 vaccination rates as of July 30, 2021. The scatter plots and correlation test results (Fig. 6) reveal significant associations between neighborhood social distancing sensitivity and these public health indicators. COVID-19 case rate, defined as the number of confirmed cases per 100,000 residents, was found to be negatively correlated with neighborhood sensitivity ($r= -0.31$), suggesting that more sensitive neighborhoods tend to engage in social distancing-compliant (or risk averse) behaviors, resulting in lower infection rates after controlling for other factors. However, we do not suggest a causal relationship. It is possible that infection rates are lower in more sensitive communities because of intrinsic neighborhood factors, such as reduced exposure densities or other social determinants of health. This finding is reinforced when looking at COVID-19 vaccination rates^49^. Neighborhood level cumulative vaccination rates (the number of fully vaccinated people normalized by total population) were found to be positively correlated with sensitivity ($r= 0.24$). Thus, more sensitive neighborhoods also tend to have high vaccination rates. These correlations demonstrate that neighborhood social distancing sensitivity can be viewed as a proxy for other public health intervention behaviors and compliance, indicating both a validation of our approach and its possible application as a predictor of compliance with other public health measures.

**Table 4:** The percentage of non-Hispanic White population for each sensitivity group by police action taken rate. Based on the citywide police action taken rate distribution, we use 0.3 and 0.6 as our thresholds, one standard deviation from the mean. The last column shows the results of t-tests for pairwise comparison.

Neighborhood Group	Police Action Taken < 0.3	Police Action Taken > 0.6	t statistics (p-value)
*SDS score* < 25th percentile	83.35% (4 zip codes)	16.28% (6 zip codes)	3.90 (0.00)***
*SDS score* 25th-50th percentile	71.40% (4 zip codes)	34.27% (33 zip codes)	1.95 (0.06)*
*SDS score* 50th-75th percentile	64.07% (7 zip codes)	39.86% (24 zip codes)	1.91 (0.07)*
*SDS score* > 75th percentile	73.01% (7 zip codes)	34.16% (6 zip codes)	4.36 (0.00)***

### City government response and potential for enforcement bias

As we have demonstrated, there are inconsistent patterns of 311 reporting across the city resulting from varying levels of sensitivity to social distancing violations. For complaint- or reporting-driven enforcement, these disparities can lead to a mis-allocation of city resources when reporting behaviors differ significantly from actual problematic conditions. In the context of local public health interventions, if more sensitive neighborhoods are more likely to complain through the 311 system, then it follows that additional resources will be allocated to these communities proportionate to the relative number of complaints. The result would be an over-allocation of resources to high sensitivity neighborhoods despite the possibility that lower sensitivity neighborhoods are at greater health risk and more in need of support. To explore the City’s response to social distancing complaints, we analyzed the relationship between neighborhood *SDS* scores and police response rates based on data included in the 311 complaint report. As shown in Fig S3, we found no statistically significant correlation between social distancing sensitivity and the rate of police response. Overall, this indicates that the likelihood of a police response to a complaint does not vary with the measured sensitivity of a neighborhood. Given the observed racial and income disparities in high and low sensitivity neighborhoods, we further segmented communities within the same *SDS* score quartiles based on the proportion of minority population to statistically test whether minority communities experience different police action rates, controlling for the sensitivity of the neighborhood. As Table 4 shows, the percentage of complaints with a police response was significantly higher in predominantly minority areas, even when holding neighborhood sensitivity constant. We explored this finding by specifying a logistic regression model based on individual complaint reports, as shown in Table S3. The results support the finding that complaints from neighborhoods with a higher percentage of Black residents are more likely to have a police response (Odds ratio == 1.74), controlling for other factors.

## Discussion

Resident reporting of complaints and problematic conditions represents an important co-production mechanism for city service delivery and management. By providing a tool for residents to report real-time issues, city agencies can use such information to improve the efficiency of resource allocation and response. More significantly, 311 systems can help to overcome systemic biases in city resource prioritization and decision-making. But such systems, when implicit and explicit biases are unknown, unaddressed, or unacknowledged, can also reinforce existing inequities and exacerbate the challenges facing vulnerable communities. In the public health context, potential bias in reporting behaviors can lead to increased health risk for under-reporting communities. We found that sensitivity to non-compliance with social distancing guidelines is lower in minority and low-income neighborhoods, resulting in fewer reported complaints than expected given levels of overcrowding in observed POIs. Neighborhoods in the lowest sensitivity quartile had, on average, a median household income of $23k less than the highest quartile, and a higher proportion of Black residents. Perhaps of interest to note, the population density in low sensitivity communities is less than half that of the most sensitive neighborhoods, suggesting density increases concerns about overcrowding, rather than acting as a desensitizing influence. Political differences also provide a stark contrast between high and low sensitivity neighborhoods: 60% of voters in the least sensitive neighborhoods vote for Republican candidates, compared to just 40% in the neighborhoods with sensitivity scores above the 75th percentile. The relationship between sensitivity and other public health metrics suggests that the behavioral response to social distancing is indicative of the local population’s perception of the risk posed by COVID-19. The least sensitive communities also had lower vaccination rates and the highest COVID-19 infection rates.

Communities where police action was taken to enforce a reported social distancing complaint had almost double the minority population compared to communities where police action was less likely, even when controlling for the level of sensitivity and reporting rates in the respective neighborhoods. It is possible that the cognizant city agencies already have an understanding of differential reporting behaviors, and that resources are deployed at higher rates to areas known to under-report. However, we see here that even in areas with similar sensitivities, the police action taken rate was higher in minority communities, which may reflect an overall greater police presence. Whatever the cause, the higher likelihood of police response may exacerbate distrust in government intervention and thus heighten an unwillingness to report problems. This concern about the nature of the city’s response to a resident report (or lack thereof) has been found with other city services^35^.

There are several limitations to this study and the interpretation of its findings. First, we are not able to directly observe individual behaviors related to social distancing or mask wearing. For instance, we do not have data on the actual distance between individuals within a POI. We therefore proceed on the assumption that increased visit density (number of persons per square foot of POI space) is associated with an increase in the likelihood of social distancing infractions. Furthermore, we do not have data on individual mask wearing behaviors, so we do not know the influence of mask use on the perception of social distancing non-compliance. Second, we are not able to de-couple the propensity to report from sensitivity. It is plausible that although someone may be more sensitive to social distancing non-compliance, they may be unwilling to report it through the 311 system. For instance, a person observing a non-complaint behavior may simply leave the establishment without reporting. Despite this, our sensitivity metric implicitly captures this reporting behavior in a way relevant to our policy context; namely, the use of reporting-based enforcement of public health policy. Whether non-reporting is caused by differences in sensitivity or reporting propensity, the potential for the mis-allocation of public health resources and enforcement remains. Third, we do not attempt to identify casualty in the relationship between social distancing sensitivity and COVID-19 infection rates. However, it is a reasonable assumption to consider that communities with lower sensitivity to social distancing are less concerned about COVID-19 risk and thus susceptible to greater transmission rates than more sensitivity neighborhoods. We control for lagging COVID-19 infection case rates to account for potential influences of perceived risk on changes in sensitivity.

Individual and collective behavioral responses to the COVID-19 pandemic have extended across a spectrum from maintaining typical mobility patterns to sheltering-in-place. While local public health policies and guidelines have nudged behavior change in certain contexts and for defined periods of time, enforcement of those policies has increasingly been centered on individual choices and actions. Our research demonstrates that a reliance on resident reporting of social distancing violations creates significant social equity concerns resulting from observed biases in reporting behaviors at the neighborhood scale.

## Materials and methods

### Data

Our primary data are POI visits provided by SafeGraph, Inc. and NYC 311 service requests available through NYC’s Open Data Portal. SafeGraph is a geospatial data company collecting anonymized cell phone location data from roughly 45 million unique devices each month, which are spatially and temporally aggregated. The SafeGraph *Places* dataset provides information on more than 9.9 million consumer Point-of-Interest (POI) locations across the US, Canada, and the United Kingdom. In addition to basic information about the POI such as unique ID, address, location, type of establishment, opening hours, and brand, the *Patterns* dataset tracks foot traffic to a given POI based on an aggregate count of the number of device visits. The activity is described by various cumulative metrics, such as the total number of devices making a stop at a particular POI, the total number of visits, the median visit duration, and median distance the device travelled based on its origin. Additionally, it contains information on the daily and hourly distribution of visits, based on the census block group (CBG) origin of the visiting devices. Importantly, the datasets do not provide information on individual devices, only counts and information aggregated by POI or CBG. SafeGraph shares its aggregated data at no cost with researchers for non-commercial purposes as part of their *SafeGraph for Academics* program and SafeGraph’s Data Consortium. The *Places* dataset used in this paper covers calendar year 2020 and includes more than 6.3 million unique POIs across the US. We filter for POIs within the administrative boundaries of the City of New York, resulting in 132,262 unique establishments of which 87,297 have detailed hourly visitor patterns data available. For each hour, we record the number of visitors and create a flag for whether the establishment was open or not. Finally, each POI location is geocoded to a specific property using a publicly-available spatial database of NYC buildings.

The other primary dataset used in this study is NYC 311 service request data. NYC 311 service requests have been collected since 2010 across more than 200 complaint and request types. Beginning on March 28, 2020, this dataset includes resident reported social distancing complaint information and city response actions at the building (tax lot) level. Each 311 complaint record contains a timestamp for the report, a geotagged location (latitude and longitude), and a property (BBL) identifier. For this study, we extract a total of 71,000 complaints related to "social distancing" and "mask wearing" based on the *"Descriptor"* column reported between March 28, 2020 and July 4, 2020.

In addition to the primary data, we retrieve multiple ancillary datasets for analysis as described in the Supplementary Materials (Table S1). NYC Primary Land Use Tax Lot Output (PLUTO) data are used to obtain land use and property category information by tax lot and zip code^50^. We use the 2019 5-year estimate U.S. Census Bureau American Community Survey (ACS) to acquire zip code level demographic, socioeconomic, housing, and job occupation characteristics^51^. In order to contextualize neighborhood social cohesion and engagement, we use nonprofit organization information with geotagged locations provided by the Urban Institute, through the National Center for Charitable Statistics^52^. Additionally, we use the standardized precinct data for the 2020 election produced by the New York Times to understand political affiliation and voting patterns across neighborhoods for the 2020 Presidential election^53^. Finally, we collect data on COVID-19-related public health indicators, specifically infection rates and vaccination rates by zip code. We use cumulative NYC COVID-19 infection rate data through July 2, 2020, including confirmed cases, deaths, positivity rates, and mortality rates, provided by the NYC Department of Health and Mental Hygiene (NYCDOH)^54^. We also obtain zip code level COVID-19 vaccination rates as of July 31, 2021, also provided by the NYCDOH^55^. All data used in this study are publicly available and acquired from open-data platforms, or available from SafeGraph as per its *SafeGraph for Academics* program.

### Building the social distancing sensitivity metric

We focus on measuring POI activity intensity (also referred to as visit density) as an indicator of overcrowding and potential social distancing non-compliance. POI activity intensity is calculated as the number of visits to a POI during a given time period divided by the POI floor area, specified as:

3 $$\begin{aligned} I_{poi_i,t} = \frac{\Sigma N_{poi_i,t}}{ a_{poi_i}} \end{aligned}$$

where $N_{poi_i,t}$ is the number of visits to POI *i* at time *t*, *t* is a given temporal unit (hourly, six-hour interval, or daily), and $a_{poi_i}$ is the floor area size of POI *i*. To maintain a scalable and uniform temporal unit that can be applied across POIs, we aggregate visits to six-hour intervals and assign a flag for whether the establishment is open or closed during the time period. Each six-hour interval is also given an indicator variable for whether a social distancing complaint was reported during that time period. The POI activity intensity when at least one social distancing complaint is reported is considered to have exceeded the complainant’s perceived threshold for when a social distancing infraction has occurred.

In order to measure social distancing sensitivity, we adapt a concept from the study of human responses to physical stimuli. We define sensitivity to social distancing non-compliance based on two sensory threshold measurements derived from Weber’s law. As Weber’s law defines an absolute threshold as the minimum intensity that results in the perception of a stimulus, we measure the absolute threshold (*A*) of social distancing compliance as the average POI activity intensity (stimulus), holding time period and floor area of the POI constant, when a 311 social distancing complaint is reported. This is mathematically defined as:

4 $$\begin{aligned} A_{poi_i} = \frac{\Sigma I_{poi_i,6h,c=1}}{n_{poi_i,c=1}} \end{aligned}$$

where $I_{poi_i,6h,c=1}$ is the count of POI visits during the six-hour interval when a social distancing complaint is reported and $n_{poi_i,c=1}$ is the number of observations with a social distancing complaint. This absolute threshold represents the average POI visit density that must be reached to result in a social distancing complaint. Together with an absolute threshold (*A*), a relative or difference threshold (*D*, the "Just Noticeable Difference threshold") represents perceptual sensitivity, which is the minimum increase in intensity of an external stimulus necessary to result in the perception of a change in intensity, proportional to the pre-existing stimulus intensity level. The difference threshold is specified as:

5 $$\begin{aligned} \Delta I = I \times D \end{aligned}$$

where $\Delta I$ is the Just Noticeable Difference (JND), *I* is the reference stimulus, and *D* is a constant, known as the Weber fraction. We use this difference threshold concept and measure *D* for each POI for each six-hour time period (the percent difference between baseline POI visit density and POI visit density when a complaint is reported) specified as:

6 $$\begin{aligned} D_{poi_i} = \frac{(A_{poi_i} - (\Sigma I_{poi_i,6h,c=0} / n_{poi_i,6h,c=0}) )}{(\Sigma I_{poi_i,6h,c=0} / n_{poi_i,6h,c=0})} \end{aligned}$$

where $A_{poi_i}$ is the absolute threshold, $I_{poi_i,6h,c=0}$ is the baseline six-hour interval POI visit density, and $n_{poi_i,6h,c=0}$ is the number of observations when no complaint is reported for a given POI. Based on the absolute threshold $(A_{poi_i})$ and the difference threshold constant $(D_{poi_i})$, sensitivity to social distancing for each POI can be determined. For example, lower absolute and relative thresholds indicate higher sensitivity in response to overcrowding and social distancing non-compliance.

In order to account for both the absolute threshold (*A*) and the difference threshold (*D*) simultaneously, we develop a unified social distancing sensitivity metric (*SDS*) based on Fechner’s Law. Fechner’s Law states that the subjective sensation intensity (*SS*) increases as the logarithm of an increase in stimulus expressed as:

7 $$\begin{aligned} SS_{poi_i} = D_{poi_i} \times log A_{poi_i} \end{aligned}$$

Lower *SS* intensity is associated with higher sensitivity, as the intensity change required to cross a threshold level of visit density deemed to be social distancing non-compliance is less. POIs with higher *SS* intensity can be considered less sensitive, as visitors tend to tolerate higher visit densities before reporting a social distancing complaint. In order to account for the relationship between *SS* and reporting sensitivity, the *SDS* value is based on a 0 to 100 scoring system and is defined mathematically as the inverse normalization of the *MinMax* re-scaled *SS* of POI visit density when a social distancing complaint is reported. Therefore, social distancing sensitivity for each POI is specified as:

8 $$\begin{aligned} SDS_{poi_i} = \left| 1 - \frac{SS_{poi_i}-min(SS_{poi})}{max(SS_{poi})-min(SS_{poi})} \right| \times 100 \end{aligned}$$

### Analyzing disparities in social distancing sensitivity and government response

Our POI level *SDS* measurement is aggregated into larger spatial units in order to estimate neighborhood sensitivity levels. We use zip code aggregation to align with the spatial resolution of demographic and socioeconomic data and COVID-19 health outcome indicators. The zip code aggregated sensitivity is defined as:

9 $$\begin{aligned} SDS_z = \frac{1}{N_z} \Sigma SDS_{poi_i} \end{aligned}$$

where $\Sigma SDS_{poi_i}$ is the sum of POI level sensitivity scores (0-100 scale) for all POIs within a given zip code *z* and $N_z$ is the number of POIs in a given zip code *z*. Although higher spatial resolutions (i.e. census tracts) can provide a more granular snapshot of heterogeneous neighborhood social distancing reporting patterns, we select the modified zip code tabulation areas defined and provided by the NYCDOH to create stable neighborhood level measurements to reduce statistical uncertainty resulting from smaller populations or the spatial distribution of POI locations. There are a total of 180 modified zip code areas across the City, less two large parks (Central Park and Prospect Park) that are excluded from the analysis.

To analyze factors associated with neighborhood disparities in behavioral responses to social distancing, we categorize zip code neighborhoods into quartiles, with "low", "medium–low", "medium–high", and "high" sensitivity labels. Neighborhood data are then aggregated for the resultant groups, which include demographic and socioeconomic characteristics, land use and built environment features, community social infrastructure, and political affiliation of voters. We apply a one-way ANOVA (Analysis of Variance) test and a Tukey’s test for posthoc analysis to examine statistically significant differences in underlying neighborhood characteristics between the four social distancing sensitivity groups. Additionally, we examine the relationships between neighborhood sensitivity scores and three indicators; COVID-19 case rates - confirmed cases per 100,000 people as of July 2, 2020; COVID-19 vaccination rates - the number of fully vaccinated people divided by the resident population as of July 30, 2021; and police action taken rates in response to social distancing complaints. These relationships are visualized using scatter plots and goodness-of-fit estimates are generated from Pearson correlation coefficients.

## Supplementary Information

Supplementary Information 1.

## Data Availability

Data needed to conduct and evaluate the analyses in the paper are described in Table S1. All processed aggregate data related to the current study may be requested from the corresponding author upon reasonable request. Mobile phone mobility data are freely available to researchers, non-profit organizations and governments through the SafeGraph Data Community (https://www.safegraph.com/academics). Other data used in this study are publicly available. New York City 311 service request data are available through the NYC Open Data Portal website (https://data.cityofnewyork.us/Social-Services/311-Service-Requests-from-2010-to-Present/erm2-nwe9). Ancillary data are publicly available. Land use and building data are available through the New York City Department of City Planning (NYCDCP) website ( https://www1.nyc.gov/site/planning/data-maps/open-data/dwn-pluto-mappluto.page). Demographic and socioeconomic data were retrieved from the US American Community Survey (ACS) administrated by Census Bureau (https://www.census.gov/programs-surveys/acs/technical-documentation/table-and-geography-changes/2019/5-year.html). Data on the 2020 Presidential election are available from the New York Times GitHub repository (https://github.com/TheUpshot/presidential-precinct-map-2020). The non-profit organization data were obtained from the Urban Institute, National Center for Charitable Statistics (https://nccs.urban.org). Data on COVID- 19 case, death, testing, and vaccination rates were obtained from the New York City Department of Health and Mental Hygiene (NYCDOH) GitHub repositories (https://github.com/nychealth/coronavirus-data and https://github.com/nychealth/covid-vaccine-data, respectively).

## Code availability

All code used to process data and perform the analysis for this study, as well as the associated results and figures were developed using Python programming language v3.6.10 and associated packages^56,57^. Maps were designed and generated with QGIS v.3.22 and Geopandas v.0.9.0 library^58,59^. All of the materials are available in the dedicated GitHub repository available at [https://github.com/UrbanIntelligenceLab/measuring-sensitivity-to-social-distancing-behavior-during-the-covid-19-pandemic] under MIT License.
