# Supplementary material for: Measuring sensitivity to social distancing behavior during the COVID-19 pandemic
Source: Sci Rep. 2022 Sep 29;12:16350. doi: 10.1038/s41598-022-20198-4 (PMC9521885; doi:10.1038/s41598-022-20198-4)
Supplement: Supplementary file 1 — Supplementary Information 1. [file 41598_2022_20198_MOESM1_ESM.pdf]

Supplementary Materials for

Measuring Sensitivity to Social Distancing Behavior during COVID-19

Constantine E. Kontokosta,*, Boyeong Hong, and Bartosz J. Bonczak

*Corresponding author. Email: ckontokosta@nyu.edu

This PDF file includes:

Figs. S1 to S3
Tables S1 to S2

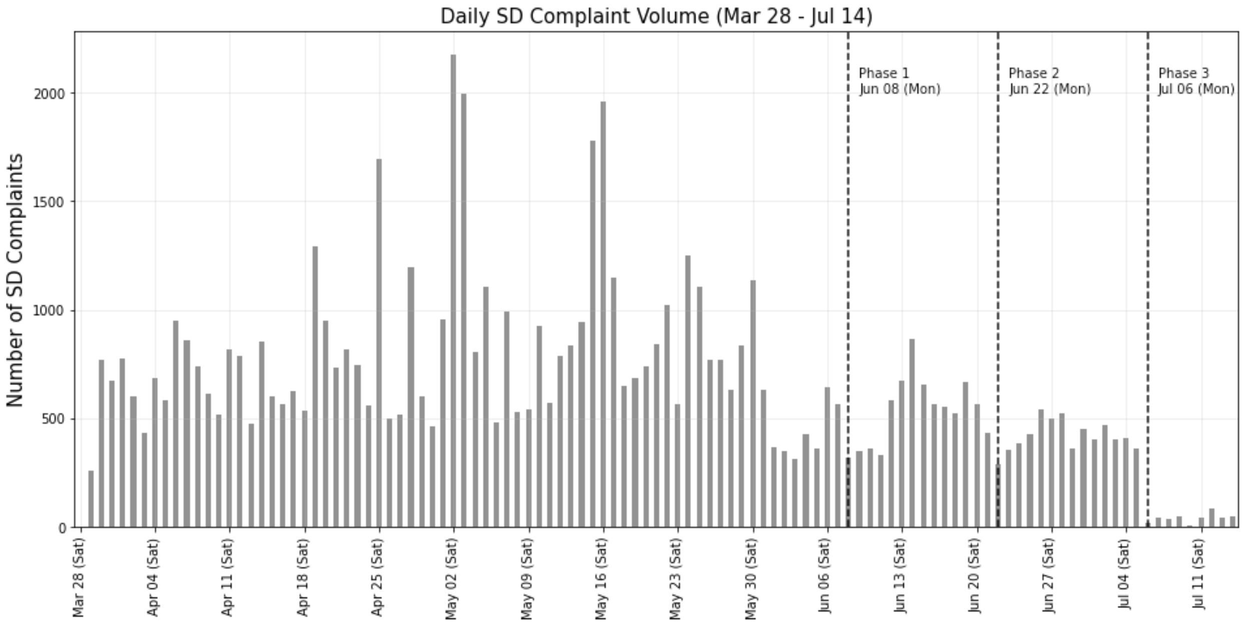

Fig. S1.
Citywide time series of social distancing complaints.

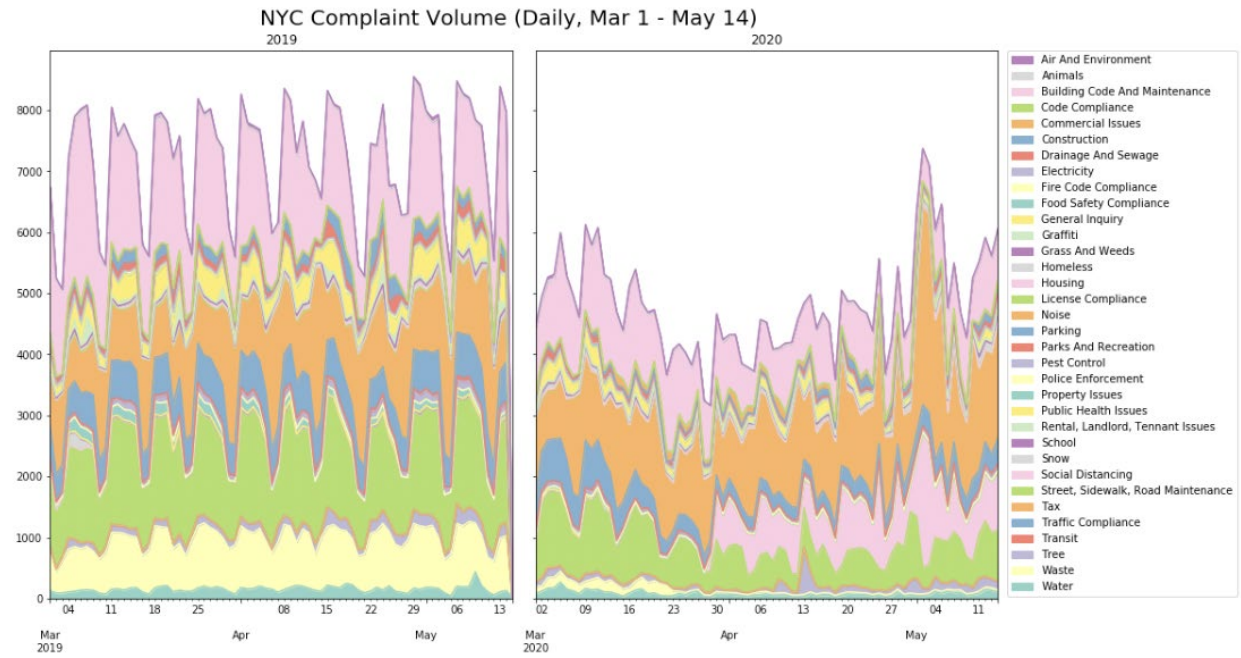

Fig. S2.
Stacked 311 complaints by type before and after the pandemic (2019 vs. 2020).

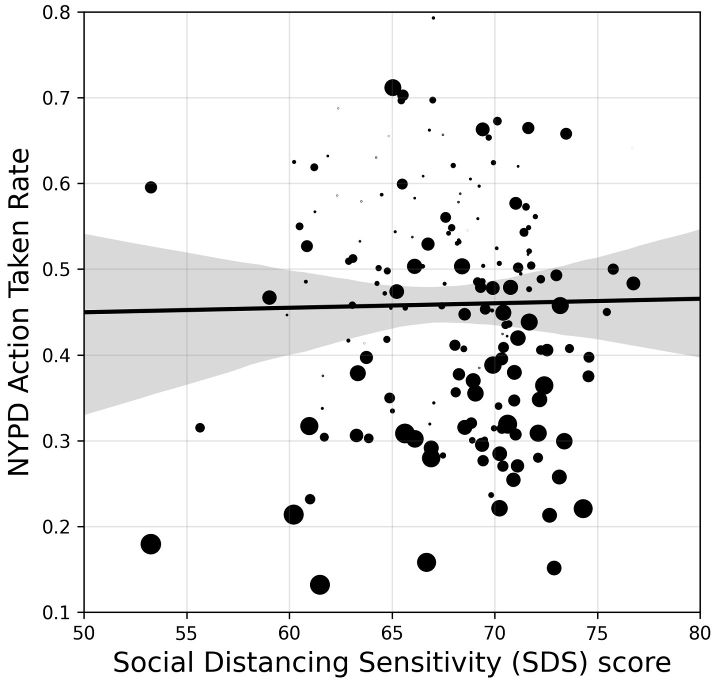

Fig. S3.

Scatter plot of neighborhood sensitivity versus police response. Proportional dot size represents the percentage of non-Hispanic White population (% of non-Hispanic White) for each zip code neighborhood.

Table S1.
Data Description.

Dataset	Time range	Resolution (spatial/temporal)	Source and description
**POI visits (Places and Patterns dataset)**	2020-03-28 ~ 2020-07-04	POI/hourly	9.9 million consumer Points-of-Interest (POI) locations and hourly foot traffic information provided by SafeGraph Inc.
**NYC 311 service request**	2020-03-28 ~ 2020-07-04	(X,Y)/second	Citizen non-emergency service request information collected and provided by the NYC311 and the NYC Department of Information Technology and Telecommunications
**NYC Primary Land Use Tax Lot Output (PLUTO)**	Updated 2020-02-24 (v20)	Parcel/NaN	Land use and building type information provided by the NYC Department of City Planning
**American Community Survey (ACS)**	2019 5-year estimate	Zip Code/NaN	Neighborhood demographic and socioeconomic characteristics from the U.S. Census Bureau
**NYT 2020 Presidential election data**	Updated 2021-03-13	Precinct/NaN	Standardized precinct-level 2020 presidential election results processed and provided by the New York Times
**Non-profit organization registration**	Updated 2018-12-13	(X,Y)/NaN	Non-profit organization locations and information provided by the Urban Institute, National Center for Charitable Statistics
**NYC COVID-19 data**	As of 2020-07-02	Zip Code/Daily	COVID-19 confirmed cases, deaths, and positivity rates provided by the NYC Department of Health and Mental Hygiene, retrieved from https://github.com/nychealth/coronavirus-data
**NYC COVID-19 vaccination data**	As of 2021-07-30	Zip Code/NaN	COVID-19 vaccination rates (partial and full) provided by the NYC Department of Health and Mental Hygiene, retrieved from https://github.com/nychealth/covid-vaccine-data

Table S2.

Results of the Tukey's multi-comparison test.

Variables	Sensitivity Groups								
		25-50	50-75	>75		50-75	>75		>75
Population density		18265.73**	27826.46***	24218.51***		9560.73	5952.78		-3607.955
Non-Hispanic White, %		1.61	7.54	14.26**		5.93	12.64*		6.72
Black, %		3.3	-6.08	-9.46		-9.39	-12.77*		-3.38
Median income, %		-4608.75	7722.72	21413.76**		12331.48	26022.52***		13691.04
College degree, %		2.35	6.07*	9.62***		3.72	7.26***		3.54
High school degree, %		-4.74*	-7.85***	-11.56***		-3.12	-6.82***		-3.7
Housing units over 750K, %		7.57	14.98**	25.41***		7.41	17.84***		10.43
Average household size		-0.3**	-0.37***	-0.49***		-0.07	-0.19		-0.12
Owner-occupied units, %		-19.67***	-23.97***	-15.98***		-4.3	3.69		7.99
Gini index		0.04**	0.03*	0.04***		-0.01	0.01		0.01
Unemployment rate, %		0.56	-0.43	-1.19		-0.99	-1.75*		-0.76
Professional and scientific workers, %	**<25**	2.24	4.80***	7.1***	**25-50**	2.56	4.87***	**50-75**	2.3
Healthcare workers, %		0.41	-1.25	-2.72***		-1.66	-3.13***		-1.47
Work from home workers, %		1.25	1.81**	3.35***		0.56	2.1***		1.54*
No health insurance, %		-0.56	0.05	-2.01*		0.61	-1.45		-2.07*
Private health insurance, %		-1.93	1.73	9.39**		3.66	11.32***		7.67
One or two family housing, %		-29.28***	-37.47***	-32.01***		-8.18	-2.73		5.45
Office area, %		5.44	5.94	11.54***		0.51	6.1		5.59
Republican voters, %		-12.96	-22.3***	-20.68**		-9.34	-7.72		1.62
Non-profit organizations, per 1K residents		0.169	-0.5	1.24**		0.33	1.07**		0.74
COVID-19 case rate, per 10K residents		-121.23	-392.99	-815.87***		-271.75	-694.63***		-422.88*
Vaccination rate, %		-4.24	3.73	7.77*		7.97*	12.01***		4.04

*** p-value < 0.01, ** p-value < 0.05, * p-value < 0.10

Table S3.

Logistic regression model results for 311 complaint police action taken rate. The dependent variable is a binary representation of police response (0=no action taken, 1=action taken). A total of 30,258 social distancing complaints are included in the model.

Dep. Variable: Police action taken (yes/no)
Model: Logit
No. Observations: 30,258
Log-Likelihood: -20373.
LLR p-value: 0.000
AUC: 0.60

Variable		Coef	Odds Ratio
**Neighborhood demographic and socioeconomic characteristics**	% of Black	0.556 (0.081)***	1.743
	% of Hispanic	-0.245 (0.076)***	0.783
	Median Income (in $10k USD)	-0.064 (0.003)***	0.938
**Complain characteristics**	Borough dummy (Bronx)	0.193 (0.050)***	1.212
	Borough dummy (Brooklyn)	0.166 (0.031)***	1.181
	Borough dummy (Queens)	0.094 (0.033)***	1.098
	Borough dummy (Staten Island)	0.014 (0.065)	1.014
	Weekend dummy	0.129 (0.025)***	1.136
	Previous complaint reporting (0=no, 1=yes)	0.164 (0.025)***	1.179
**Types of POI**	Beauty and personal cares	-0.058 (0.031)*	0.944
	Parks	0.124 (0.044)***	1.131
	Banks	0.029 (0.034)	1.029
	Food and drink places	-0.029 (0.024)	0.971
	Grocery stores	0.110 (0.027)***	1.117
	Gyms	0.072 (0.039)*	1.075
	Hospitals and clinics	-0.071 (0.029)**	0.931
	Daycare or education centers	-0.098 (0.044)**	0.907
	Transportation facilities	-0.575 (0.154)***	0.563
	Religious facilities	0.053 (0.091)	1.055
**Neighborhood social distancing sensitivity**	Low sensitivity neighborhood dummy	-0.344 (0.073)***	0.709
	High sensitivity neighborhood dummy	-0.028 (0.049)	1.029
**Interaction terms**	Black X low sensitivity neighborhood	0.003 (0.001)**	1.003
	Black X high sensitivity neighborhood	-0.0002 (0.002)	1.000
	Hispanic X low sensitivity neighborhood	0.010 (0.002)***	1.010
	Hispanic X high sensitivity neighborhood	0.001 (0.002)	1.001

Note: Standard errors in parentheses. *** p-value < 0.01, ** p-value < 0.05, * p-value < 0.10
